# Protective Effects of Dietary Supplements Containing Probiotics, Micronutrients, and Plant Extracts Against Lead Toxicity in Mice

**DOI:** 10.3389/fmicb.2018.02134

**Published:** 2018-09-11

**Authors:** Qixiao Zhai, Liu Yang, Jianxin Zhao, Hao Zhang, Fengwei Tian, Wei Chen

**Affiliations:** ^1^State Key Laboratory of Food Science and Technology, Jiangnan University, Wuxi, China; ^2^School of Food Science and Technology, Jiangnan University, Wuxi, China; ^3^International Joint Research Laboratory for Probiotics, Jiangnan University, Wuxi, China; ^4^National Engineering Research Center for Functional Food, Jiangnan University, Wuxi, China; ^5^Beijing Innovation Center of Food Nutrition and Human Health, Beijing Technology and Business University (BTBU), Beijing, China

**Keywords:** lead toxicity, dietary supplements, *Lactobacillus plantarum*, micronutrients, plant extracts

## Abstract

Lead (Pb) intoxication is a serious food safety issue, and the development of relevant dietary strategies is an area of ongoing research. In this study, two different dietary supplements were designed and evaluated for their effects against Pb toxicity in mice. Dietary supplement A contained grape seed extract, tea polyphenols and *Lactobacillus plantarum* CCFM8661, and dietary supplement B contained vitamin C, calcium carbonate, zinc acetate, and *L. plantarum* CCFM8661. The results showed that both dietary supplements could effectively decrease Pb levels, protect aminolevulinic acid dehydratase, superoxide dismutase and catalase activities and recover glutathione, zinc protoporphyrin and malondialdehyde levels in tissues and blood of mice. A step-through passive avoidance task confirmed that the dietary supplements could recover the learning and memory capacities of Pb-exposed mice. The protective effects of both dietary supplements to alleviate oxidative stress and cognitive impairments were superior to the chelator treatment. Administration of the dietary supplements during Pb exposure offered more significant protection than administration after Pb exposure. Animal safety evaluation also indicated that these dietary supplements barely induced side effects in the mice. This study provides evidence that dietary supplements containing probiotics, micronutrients, and plant extracts can be considered a new dietary strategy against Pb toxicity.

## Introduction

Lead (Pb) is a pollutant widely distributed in the environment and food chain. Once accumulated in the human body, Pb and its compounds induce oxidative stress and disrupt the metabolism of essential metals (Gurer and Ercal, 2000; Dobrakowski et al., 2017), which in turn leads to acute or chronic toxic effects on the nervous (Winder et al., 1982; Cecil et al., 2008), hematic (Redig et al., 1991; Ho and Ho, 1997), hepatic (Farmand et al., 2005; Xia et al., 2010), urinary (Loghman-Adham, 1997; Fels et al., 1998), and cardiovascular systems (Freeman, 1965). Pb intoxication is related with clinical symptoms including mental hypomnesis, anemia, tubular atrophy, and even death. Industrial development has caused the number of Pb poisoning cases to increase in recent years. A Pb water pollution incident occurring in Flint, MI, United States in 2015 led to a sharp increase in the blood Pb levels of local children (Hannaattisha et al., 2016). Another population-based study showed that the blood Pb levels of children living in Guiyu, one of the most seriously heavy metal-polluted regions of China, have reached 7.06 mg/dL (Guo et al., 2014).

The most common therapeutic strategy against Pb poisoning is to promote the metal’s excretion by using chelating agents such as ethylenediaminetetraacetic acid (EDTA) and dimercaptosuccinic acid (DMSA). However, these chelators have a range of side effects (Paternain et al., 1990; Jorgensen, 1993; Porru and Alessio, 1996; Chen et al., 1999) and are not suitable for high-dose and long-term treatment. Thus, increasing attention is being paid to seeking safe dietary supplements, such as micronutrients, plant extracts and probiotics, for the intervention and alleviation of Pb intoxication.

Supplementation with micronutrients including essential trace elements and vitamins has been reported to provide protection against Pb toxicity (Cerklewski and Forbes, 1976; Shaban and Said, 2011; Basha et al., 2012). Metals such as calcium (Ca) and zinc (Zn) have similar chemical properties to Pb and can compete for the binding sites of metal absorptive and enzymatic proteins in the gut, blood, and tissues of humans (Basha et al., 2012). Zn supplementation also induces the biosynthesis of metallothioneins, proteins involved in the detoxification of heavy metals (Baltaci et al., 2018). Recent studies showed that the intake of Ca and Zn can reduce the level of Pb metal in the blood and bone of pregnant women (Gulson et al., 2004) and children (Cantoral et al., 2015). Vitamin C is a non-enzymatic antioxidant and a potential chelator of Pb (Goyer and Cherian, 1979). Daily supplementation of vitamin C has been reported to reduce blood Pb levels and alleviate lipid peroxidation in erythrocytes of smokers and Pb-exposed workers (Dawson et al., 1999; Rendón-Ramírez et al., 2014).

An increasing number of studies have shown that plant extracts with antioxidative stress properties can effectively alleviate Pb toxicity (Hsu and Guo, 2002; Jiao et al., 2011). Grape seed extract, which is rich in procyanidins, could recover lesions in the cardiovascular system induced by Pb exposure and inhibit alterations in the levels of adrenaline, 5-hydroxytryptamine and glutamic pyruvic transaminase (ALT) in the brain and liver of rats (Waggas, 2012). Another common plant extract, tea polyphenols, was reported to prevent Pb-induced dysfunctions in the brain, blood and liver of humans and animals (Hamed et al., 2010; Mehana et al., 2012). Catechins, one of the main constituents of tea polyphenols, were able to ameliorate cytotoxicity in renal and nerve cells under Pb exposure (Chen et al., 2002, 2003).

Apart from the above-mentioned dietary supplements, recent reports revealed that probiotics were capable of binding Pb *in vitro* and diminishing oxidative stress *in vivo*, indicating their potential against Pb toxicity (Halttunen et al., 2007). In our previous studies, *Lactobacillus plantarum* CCFM8661 was screened out for its good tolerance and ability to bind to Pb (Tian et al., 2012; Yin et al., 2016). Further animal experiments confirmed that this strain decreased Pb levels in blood and tissues of mice, and offered significant protection during the recovery from Pb-induced oxidative stress and histopathological changes in mice (Tian et al., 2012).

Based on these studies, it is believed that the supplements of specific dietary components can be effective antagonists to Pb intoxication. Further studies are needed to develop relevant therapeutic dietary strategies. We noticed that previous reports mainly focused on a single component and that the synergistic effect of micronutrients, plant extracts and probiotics against Pb toxicity was rarely investigated. Therefore, in the present study, two kinds of multiple dietary supplements were designed and their safety and protective effects against Pb toxicity were evaluated in mice.

## Materials and Methods

### Chemicals and Reagents

Dimercaptosuccinic acid was purchased from New Asia Pharmaceutical Company (Shanghai, China). Grape seed extract was purchased from Jianfeng Natural Product Research and Development Company (Tianjin, China). Tea polyphenols were purchased from Lvkang Natural Products Company (Nanchang, China). Microcrystalline cellulose, low-substituted hydroxypropyl cellulose and silicon dioxide were purchased from Tianzheng Pharmaceutical Excipients Company (Xian, China). Kits used to measure the levels of malondialdehyde (MDA), glutathione (GSH), albumin (ALB), blood urea nitrogen (BUN), creatinine (CRE), total cholesterol (TCHO), triglyceride (TG), and total protein (TP) and the activities of superoxide dismutase (SOD), catalase (CAT), ALT, and glutamic oxalacetic transaminase (AST) were purchased from the Jiancheng Bioengineering Institute (Nanjing, China). Kits used to measure the levels of zinc protoporphyrin (ZPP) and the activities of δ-aminolevulinic acid dehydratase (ALAD) were purchased from the Chuanxiang Bioengineering Company (Wuhan, China). Pb acetate and all other analytical laboratory chemicals and reagents were purchased from Sinopharm Chemical Reagent Company (Shanghai, China).

### Bacterial Strains and Culture

*Lactobacillus plantarum* CCFM8661 were obtained from the Culture Collections of Food Microbiology, Jiangnan University (Wuxi, China). The bacteria were cultured in de Man, Rogosa and Sharpe broth (Hopebio Company, Qingdao, China) at 37°C for 18 h. The bacterial cells were freeze-dried as previously reported (Zhai et al., 2014) with the protectant replaced by trehalose.

### Design of the Dietary Supplements

The components of the two different dietary supplements are listed in **Table 1**. Microcrystalline cellulose, low-substituted hydroxypropyl cellulose and silicon dioxide served as filler, disintegrant and lubricant, respectively (Liu et al., 2009; Mahmoud et al., 2009). The doses of micronutrients, plant extracts, and probiotics were selected based on previous studies to provide protective effects against Pb toxicity (Liao et al., 2008; Mehana et al., 2012; Tian et al., 2012; Waggas, 2012). Dietary supplements A (DSA) and B (DSB) were designed to evaluate the protection of a plant extracts-probiotics mixture and a micronutrients-probiotics mixture, respectively. Both supplements were pressed into tablets of 240–260 mg each.

**Table 1:** Components of the two dietary supplements.

Types	Components	Content(%)
Dietary supplement A (DSA)	Microcrystalline cellulose	48.9
	Low-substituted hydroxypropyl cellulose	5
	Silicon dioxide	0.1
	Grape seed extract	3.6
	Tea polyphenols	2.4
	Freeze-dried bacterial powder	40
	Total	100
Dietary supplement B (DSB)	Microcrystalline cellulose	46.5
	Low-substituted hydroxypropyl cellulose	5
	Silicon dioxide	0.1
	Vitamin C	1.6
	Calcium carbonate	6.4
	Zinc acetate	0.4
	Freeze-dried bacterial powder	40
	Total	100

Based on our previous study, no significant alterations in the levels of procyanidins, catechins, and vitamin C could be observed after tableting, whereas the number of living bacterial cells of *L. plantarum* CCFM8661 decreased to approximately 8 × 109 cfu/g tablet (Liu et al., 2016).

### Animals and Design of the Pb Intoxication Experiment

Six-week-old male C57black/6 mice (weighing 25–30 g) obtained from the Shanghai Laboratory Animal Center (Shanghai, China) were used in the experiments. Mice were maintained in a room kept on a 12-h light/dark cycle at a constant temperature and humidity and had free access to standard commercial mouse food and distilled water. All animal experiments described herein were approved by the Ethics Committee of Jiangnan University, China (JN No. 20150811-1211-51) and performed in accordance with the guidelines set by the European Community (directive 2010/63/EU).

As shown in **Table 2**, 120 mice were randomly divided into three groups. The intervention and therapy groups were designed to evaluate the protective effects of the dietary supplements against Pb toxicity during and after chronic exposure, respectively. The DMSA groups were set as a drug control. The intervention and therapy groups were divided into five subgroups, and an oral dose of Pb at 1 g/L of drinking water was used to model chronic exposure (Tangpong et al., 2010).

**Table 2:** Animal experimental design.

Groups		Treatment on the week(s) indicated		
		0–8 weeks	9 weeks	10–16 weeks
Intervention groups	Control	PW + PBS	PW + PBS	–
	Pb only	Pb only + PBS	PW + PBS	–
	Pb + DSA	Pb + DSA + PBS	PW + PBS	–
	Pb + DSB	Pb + DSB + PBS	PW + PBS	–
	Pb + Excipients	Pb + Excipients + PBS	PW + PBS	–
DMSA groups	High-dose DMSA	Pb + PBS	PW + H-DMSA + PBS	–
	Low-dose DMSA	Pb + PBS	PW + L-DMSA + PBS	–
Therapy groups	Control	PW	PW + PBS	PW + PBS
	Pb only	Pb	PW + PBS	PW + PBS
	Pb + DSA	Pb	PW + DSA + PBS	PW + DSA + PBS
	Pb + DSB	Pb	PW + DSB + PBS	PW + DSB + PBS
	Pb + Excipients	Pb	PW + Excipients + PBS	PW + Excipients + PBS

*PBS, 0.3 sterile PBS solution once daily via gavage; PW, plain water for drinking; Pb, (CH_3_COO)_2_Pb⋅3H_2_O at 1 g Pb/L in drinking water; DSA + PBS, half a tablet of dietary supplement A in 0.3 mL sterile PBS solution once daily via gavage; DSB + PBS, half a tablet of dietary supplement B in 0.3 mL sterile PBS solution once daily via gavage; Excipients + PBS, 62.5 mg microcrystalline cellulose, 6.25 mg low-substituted hydroxypropyl cellulose, 0.25 mg silicon dioxide, and 40 mg trehalose in 0.3 mL sterile PBS solution once daily via gavage; H-DMSA + PBS, 3 mg DMSA in 0.3 mL sterile PBS solution once daily via gavage; L-DMSA + PBS, 1.5 mg DMSA in 0.3 mL sterile PBS solution once daily via gavage. Mice were fasted for 12 h before Pb exposure and final sacrifice. The mice in the intervention groups were sacrificed at the end of the 9th week.*

At the end of the experiment, mice underwent a step-through passive avoidance task and were then sacrificed under light ether anesthesia. A portion of blood was collected in anticoagulant tubes for routine hematological examination. The remaining blood was centrifuged to obtain serum and stored at −80°C. The liver, whole kidneys, and brains were weighed and stored in metal-free Eppendorf tubes at −80°C for the biochemical assays and estimation of Pb levels.

### Step-Through Passive Avoidance Task

The step-through task was performed on the last 2 days of the gavage according to previous reports (Lu et al., 2011; Ferlemi et al., 2014). The step-through latency (time taken to completely enter the darkened chamber) was recorded to evaluate the learning and memory of mice.

### Determination of Pb in Tissues and Blood

The liver, whole kidneys, brain, and blood samples were digested in concentrated HNO_3_ with a Microwave Digestion System (MARS; CEM, United Kingdom). The contents of Pb in tissues and blood were determined by using flame or graphite furnace atomic absorption spectrophotometry (Spectr AAS or AA; Varian, United States).

### Determination of Biochemical Indicators in Tissues and Serum

The levels of MDA and GSH and the activities of SOD and CAT in tissues were measured with kits purchased from Jiancheng Bioengineering Institute (Nanjing, China). The levels of ZPP and the activities of ALAD in serum were measured with kits obtained from Chuanxiang Bioengineering Company (Wuhan, China).

### Determination of Red Blood Cell and Hemoglobin Levels in the Blood

The levels of red blood cells (RBCs) and hemoglobin in blood were measured using an automated hematology analyzer (XE-2100, Sysmex, Japan).

### Animals and Design of the Safety Evaluation Experiment

The safety of the two dietary supplements was evaluated by an acute toxicity test and a 30-day feeding trial in mice (Silva et al., 2011). The animal experiments were approved by the Ethics Committee of Jiangnan University, China (JN No.20150519-0704-34 and JN No 20150707-0820-47).

For the acute toxicity test, 60 C57black/6 mice (half male and half female, 3 weeks of age on arrival) were obtained from the Shanghai Laboratory Animal Center (Shanghai, China). After acclimation, mice were randomly divided into three groups: control group (receiving a single oral dose of PBS at 0.4 mL/20 g body weight via gavage), DSA group (receiving a single oral dose of dietary supplement A at 4 g/20 g body weight via gavage) and DSB group (receiving a single oral dose of dietary supplement B at 4 g/20 g body weight via gavage). Male and female mice were housed in separate cages. After the oral administration, mice were kept for 2 weeks and their body weight, food consumption, and behavior were recorded. At the end of the experiment, mice were sacrificed, and the histopathological alterations of liver, kidneys, and spleen were observed.

For the 30-day feeding trial, 140 C57black/6 mice (half male and half female, 3 weeks of age on arrival) were obtained from the Shanghai Laboratory Animal Center (Shanghai, China). After acclimation, mice were randomly divided into seven groups: control group (receiving 0.2 mL PBS via gavage each day) and low-dose DSA group, mid-dose DSA group, high-dose DSA group, low-dose DSB group, mid-dose DSB group, and high-dose DSB group. Mice in the latter six groups received dietary supplement A or B at 10, 15, and 20 g/kg body weight each day, respectively. The functional components of the dietary supplements including probiotics, micronutrients, and plant extracts were given via gavage in 0.2 mL PBS, and the filler, disintegrant, and lubricant were mixed into the feed of the mice. Male and female mice were housed in separate cages. Their body weight, food consumption, and behavior were recorded during the experiment. After sacrifice, the routine hematological indicators were measured using an automated hematology analyzer (XE-2100, Sysmex, Japan). The biochemical parameters in blood including ALB, ALT, AST, BUN, CRE, TCHO, TG, and TP were determined using commercial kits. The histopathological alterations of liver, kidneys, and spleen were also observed.

### Statistical Analysis

All data are presented as the mean ± standard deviation and were analyzed by one-way analysis of variance (ANOVA) and Duncan *post hoc*-test. The data were considered to show statistical significance at a *P*-value of 0.05 or less.

## Results

### Pb Levels in Tissues and Blood of Mice

As shown in **Table 3**, Pb levels in the liver, kidneys, brain, and blood of Pb-treated mice were significantly higher than those of the control group (*P* < 0.05). Oral administration of both dietary supplements decreased the levels of Pb in tissues and blood (*P* < 0.05), although the protection was not as obvious as that with the DMSA treatments. Dietary supplements induced more marked reduction in Pb levels in the liver and blood of mice in the intervention groups than in the therapy groups.

**Table 3:** Effects of dietary supplements on lead levels in the tissues and blood of mice.

Groups		Pb levels in liver (μg/g wet tissues)	Pb levels in kidneys (μg/g wet tissues)	Pb levels in brain (μg/g wet tissues)	Pb levels in blood (μg/L)
Intervention groups	Control	0.37 ± 0.02^f^	0.48 ± 0.09^d^	0.33 ± 0.05^d^	38.60 ± 6.59^f^
	Pb only	2.54 ± 0.07^a^	4.79 ± 0.28^a^	1.91 ± 0.13^a^	380.40 ± 28.01^a^
	Pb + DSA	1.44 ± 0.10^c^	2.74 ± 0.29^b^	1.44 ± 0.15^b^	187.67 ± 17.40^cd^
	Pb + DSB	1.40 ± 0.12^c^	2.41 ± 0.13^b^	1.22 ± 0.10^b^	169.22 ± 5.27^d^
	Pb + Excipients	2.51 ± 0.20^a^	4.57 ± 0.57^a^	2.07 ± 0.17^a^	364.37 ± 18.14^a^
DMSA groups	High-dose DMSA	0.82 ± 0.13^e^	1.47 ± 0.14^c^	0.74 ± 0.10^c^	88.23 ± 12.43^e^
	Low-dose DMSA	1.07 ± 0.06^d^	1.80 ± 0.25^c^	0.94 ± 0.12^c^	105.05 ± 6.25^e^
Therapy groups	Control	0.39 ± 0.03^f^	0.53 ± 0.04^d^	0.39 ± 0.05^d^	35.14 ± 10.40^f^
	Pb only	2.51 ± 0.05^a^	5.09 ± 0.46^a^	1.91 ± 0.06^a^	379.40 ± 16.07^a^
	Pb + DSA	1.66 ± 0.06^b^	2.89 ± 0.40^b^	1.44 ± 0.09^b^	213.05 ± 11.56^b^
	Pb + DSB	1.64 ± 0.16^b^	2.79 ± 0.34^b^	1.33 ± 0.18^b^	205.34 ± 13.58^bc^
	Pb + Excipients	2.43 ± 0.08^a^	4.50 ± 0.53^a^	1.98 ± 0.17^a^	364.60 ± 9.93^a^

*Values are for 10 mice per group. Abbreviations of group names are as in **Table 2**. ^a–f^Statistically significant differences (P < 0.05) within each group comparison.*

### ALAD Activities in Serum of Mice

**FIGURE 1:**
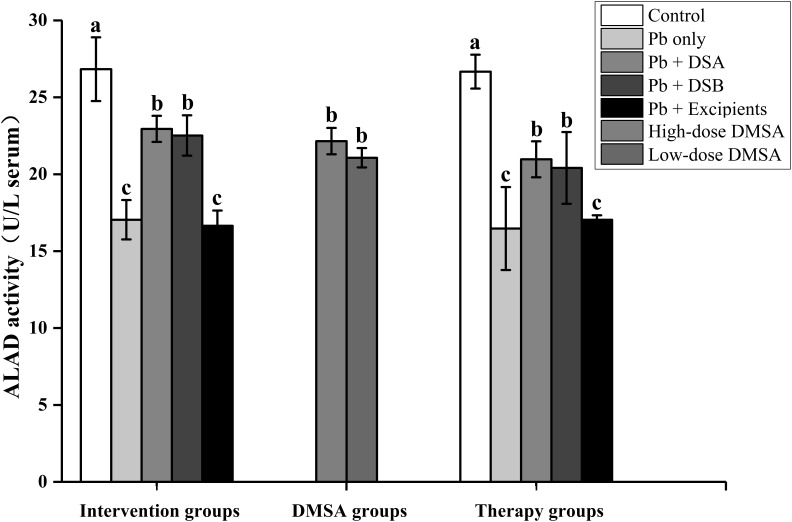
Effects of dietary supplements on ALAD activities in the serum of mice. Values are for 10 mice per group. Letters a to c indicate statistically significant differences (*P* < 0.05) within each group comparison. Abbreviations of group names are as in **Table 2**.

Lead exposure induced a significant decrease in ALAD activities in the serum of mice (**Figure 1**). Oral administration of both DSA and DSB recovered the ALAD activities (*P* < 0.05). No marked difference in the activities of this enzyme could be observed after the intake of dietary supplements between the intervention and therapy groups.

### ZPP Levels in Serum of Mice

**FIGURE 2:**
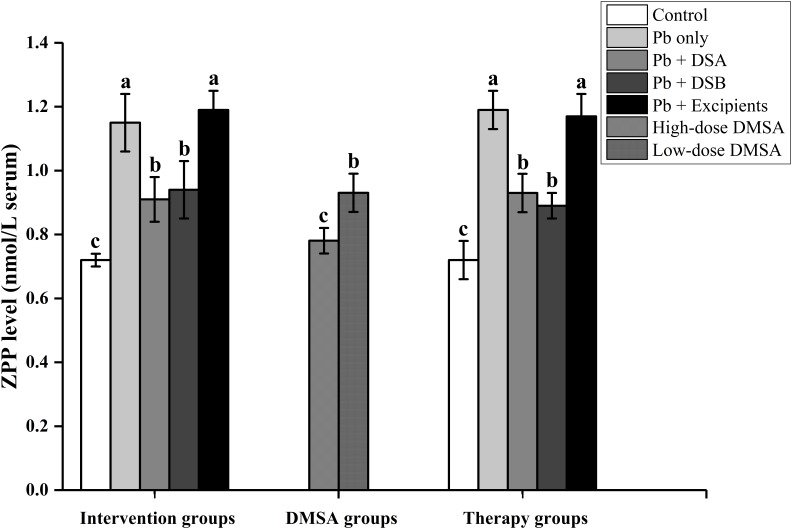
Effects of dietary supplements on ZPP levels in the serum of mice. Values are for 10 mice per group. Letters a to c indicate statistically significant differences (*P* < 0.05) within each group comparison. Abbreviations of group names are as in **Table 2**.

Supplementation of both DSA and DSB inhibited the Pb-induced increase of ZPP levels in the serum of mice (**Figure 2**, *P* < 0.05). However, the protection was not as obvious as that with the DMSA treatments.

### Activities of SOD and CAT and the Levels of MDA and GSH in Tissues of Mice

As shown in **Tables 4**–**6**, the activities of SOD and CAT were inhibited in the livers, kidneys, and brains of Pb-treated mice (*P* < 0.05), except for undetected CAT activities in the brains. Pb exposure also induced alterations in the levels of GSH and MDA in these tissues of mice. Oral administration of both DSA and DSB recovered the changes in these biochemical indicators (*P* < 0.05), and the protection was more significant than that offered by the DMSA treatment.

**Table 4:** Effects of dietary supplements on the activities of SOD and CAT and the levels of MDA and GSH in the livers of mice.

Groups		SOD (U/mg protein)	CAT (U/mg protein)	MDA (nmol/mg protein)	GSH (μmol/g protein)
Intervention groups	Control	6.57 ± 1.18^a^	334.24 ± 16.00^b^	1.18 ± 0.39^b^	2.84 ± 0.16^b^
	Pb only	3.32 ± 0.67^b^	206.90 ± 12.52^d^	2.10 ± 0.41^a^	1.44 ± 0.34^d^
	Pb + DSA	7.08 ± 0.65^a^	390.73 ± 22.11^a^	1.12 ± 0.14^b^	3.94 ± 0.60^a^
	Pb + DSB	6.27 ± 0.13^a^	380.41 ± 38.87^a^	1.23 ± 0.34^b^	3.61 ± 0.40^a^
	Pb + Excipients	3.20 ± 0.72^b^	211.77 ± 14.32^d^	2.12 ± 0.28^a^	1.54 ± 0.09^d^
DMSA groups	High-dose DMSA	4.18 ± 0.46^b^	280.27 ± 13.17^c^	1.49 ± 0.08^b^	2.30 ± 0.18^c^
	Low-dose DMSA	4.09 ± 0.63^b^	265.83 ± 14.92^c^	1.57 ± 0.13^b^	2.14 ± 0.14^c^
Therapy groups	Control	6.34 ± 0.86^a^	331.00 ± 32.28^b^	1.03 ± 0.21^b^	2.82 ± 0.16^b^
	Pb only	2.96 ± 0.40^b^	223.47 ± 32.88^d^	2.45 ± 0.28^a^	1.64 ± 0.23^d^
	Pb + DSA	6.57 ± 0.68^a^	377.12 ± 20.96^a^	1.21 ± 0.04^b^	3.53 ± 0.02^a^
	Pb + DSB	6.21 ± 0.98^a^	393.25 ± 18.18^a^	1.37 ± 0.16^b^	3.73 ± 0.19^a^
	Pb + Excipients	3.12 ± 0.61^b^	213.97 ± 26.07^d^	2.53 ± 0.52^a^	1.55 ± 0.37^d^

*Values are for 10 mice per group. Abbreviations of group names are as in **Table 2**. ^a–d^Statistically significant differences (P < 0.05) within each row comparison.*

**Table 5:** Effects of dietary supplements on the activities of SOD and CAT and the levels of MDA and GSH in the kidneys of mice.

Groups		SOD (U/mg protein)	CAT (U/mg protein)	MDA (nmol/mg protein)	GSH (μmol/g protein)
Intervention groups	Control	10.77 ± 0.16^a^	141.61 ± 5.54^a^	2.72 ± 0.11^bc^	10.13 ± 0.46^a^
	Pb only	5.95 ± 0.34^b^	92.57 ± 7.22^c^	4.04 ± 0.17^a^	5.61 ± 0.32^b^
	Pb + DSA	10.21 ± 2.69^a^	152.49 ± 14.76^a^	2.20 ± 0.36^c^	11.10 ± 1.37^a^
	Pb + DSB	10.71 ± 0.84^a^	153.42 ± 7.90^a^	2.38 ± 0.25^c^	10.32 ± 0.89^a^
	Pb + Excipients	5.67 ± 0.24^b^	90.02 ± 14.94^c^	4.08 ± 0.44^a^	5.54 ± 0.22^b^
DMSA groups	High-dose DMSA	7.42 ± 0.46^b^	124.41 ± 9.06^b^	3.18 ± 0.15^b^	6.77 ± 0.18^b^
	Low-dose DMSA	7.16 ± 0.54^b^	116.46 ± 6.73^b^	3.32 ± 0.11^b^	6.53 ± 0.39^b^
Therapy groups	Control	11.04 ± 0.80^a^	141.11 ± 6.79^a^	2.78 ± 0.10^b,c^	9.99 ± 1.01^a^
	Pb only	5.48 ± 1.22^b^	97.76 ± 2.77^c^	4.58 ± 0.59^a^	5.58 ± 0.23^b^
	Pb + DSA	9.78 ± 1.63^a^	147.63 ± 8.45^a^	2.39 ± 0.46^c^	10.30 ± 0.81^a^
	Pb + DSB	10.39 ± 0.52^a^	140.58 ± 3.12^a^	2.51 ± 0.42^c^	10.00 ± 0.77^a^
	Pb + Excipients	6.16 ± 0.17^b^	97.68 ± 5.34^c^	4.60 ± 0.50^a^	5.79 ± 0.24^b^

*Values are for 10 mice per group. Abbreviations of group names are as in **Table 2**. ^a–c^Statistically significant differences (P < 0.05) within each row comparison.*

**Table 6:** Effects of dietary supplements on SOD activity and the levels of MDA and GSH in the brains of mice.

Groups		SOD (U/mg protein)	MDA (nmol/mg protein)	GSH (μmol/g protein)
Intervention groups	Control	8.46 ± 0.47^a^	6.76 ± 0.67^c^	11.57 ± 0.61^a^
	Pb only	3.13 ± 0.38^d^	11.13 ± 0.30^a^	6.49 ± 0.36^c^
	Pb + DSA	6.24 ± 0.38^b,c^	6.63 ± 0.64^c^	12.34 ± 0.47^a^
	Pb + DSB	6.53 ± 0.28^b^	7.31 ± 0.23^c^	11.58 ± 0.41^a^
	Pb + Excipients	3.17 ± 0.79^d^	11.13 ± 0.69^a^	6.51 ± 0.26^c^
DMSA groups	High-dose DMSA	6.01 ± 0.28^b,c^	9.49 ± 1.31^b^	9.53 ± 0.10^b^
	Low-dose DMSA	5.28 ± 0.51^c^	9.47 ± 0.74^b^	8.94 ± 0.43^b^
Therapy groups	Control	9.05 ± 1.50^a^	6.52 ± 0.31^c^	11.28 ± 0.33^a^
	Pb only	4.04 ± 0.38^d^	12.05 ± 2.01^a^	6.56 ± 1.20^c^
	Pb + DSA	6.17 ± 0.17^b,c^	7.35 ± 0.72^c^	12.61 ± 0.77^a^
	Pb + DSB	5.92 ± 0.61^b,c^	7.50 ± 0.55^c^	12.61 ± 2.33^a^
	Pb + Excipients	3.95 ± 0.61^d^	12.29 ± 0.47^a^	6.43 ± 0.55^c^

*Values are for 10 mice per group. Abbreviations of group names are as in **Table 2**. ^a–d^Statistically significant differences (P < 0.05) within each row comparison.*

### Levels of RBCs and Hemoglobin in the Blood of Mice

Lead exposure decreased the levels of RBCs and hemoglobin in the serum of mice (**Table 7**). Both DSA and DSB supplementation recovered the alterations in these hematological indicators (*P* < 0.05), and the protection was as obvious as that with DMSA treatment.

**Table 7:** Effects of dietary supplements on red blood cell and hemoglobin levels in the blood of mice.

Groups		Red blood cells (10^12^/L)	Hemoglobin (g/L)
Intervention groups	Control	10.74 ± 0.40^a^	160.33 ± 3.21^a^
	Pb only	9.73 ± 0.04^b^	130.67 ± 2.52^c^
	Pb + DSA	10.57 ± 0.45^a^	151.67 ± 6.43^a,b^
	Pb + DSB	11.02 ± 0.13^a^	154.33 ± 10.79^a,b^
	Pb + Excipients	9.57 ± 0.42^b^	130.00 ± 4.00^c^
DMSA groups	High-dose DMSA	10.90 ± 0.21^a^	152.33 ± 4.04^ab^
	Low-dose DMSA	10.75 ± 0.21^a^	149.67 ± 3.79^b^
Therapy groups	Control	10.69 ± 0.28^a^	159.67 ± 3.06^a^
	Pb only	9.41 ± 0.20^b^	124.67 ± 2.31^c^
	Pb + DSA	10.56 ± 0.24^a^	147.67 ± 4.93^b^
	Pb + DSB	10.66 ± 0.30^a^	148.00 ± 4.58^b^
	Pb + Excipients	9.47 ± 0.33^b^	126.67 ± 1.53^c^

*Values are for 10 mice per group. Abbreviations of group names are as in **Table 2**. ^a–c^Statistically significant differences (P < 0.05) within each row comparison.*

### Learning and Memory Ability of Mice

Compared with control groups, Pb exposure induced marked deficits in the learning and memory abilities of mice (**Table 8**). Both dietary supplements significantly reduced the step-through latency time, and their effects were superior to that of the DMSA treatment (*P* < 0.05). We also noticed that both DSA and DSB administration showed better protection in the intervention groups than that in the therapy groups.

**Table 8:** Effects of dietary supplements on the step-through latency of mice.

Groups		Latency/second
Intervention groups	Control	330.79 ± 26.45^a^
	Pb only	76.30 ± 4.71^d^
	Pb + DSA	211.28 ± 35.47^b^
	Pb + DSB	223.05 ± 19.95^b^
	Pb + Excipients	74.65 ± 1.60^d^
DMSA groups	High-dose DMSA	118.98 ± 8.73^c^
	Low-dose DMSA	120.79 ± 16.58^c^
Therapy groups	Control	353.21 ± 11.76^a^
	Pb only	73.43 ± 0.75^cd^
	Pb + DSA	142.70 ± 10.00^c^
	Pb + DSB	134.41 ± 5.21^c^
	Pb + Excipients	72.93 ± 3.67^d^

*Values are for eight mice per group. Abbreviations of group names are as in **Table 2**. ^a–d^Statistically significant differences (P < 0.05) within each row comparison.*

### Safety Evaluation of the Dietary Supplements

Both the acute toxicity test and 30-day feeding trial showed that the supplementation of DSA and DSB caused no significant alterations in the body weight, food consumption, and behavior of mice (data not shown). No changes in the levels of RBCs, hemoglobin, leukocytes, ALB, ALT, AST, BUN, CRE, TCH, TG, and TP in the blood could be observed after the intake of the dietary supplements (**Supplementary Tables S1**, **S2**). The supplements did not induce changes in organ coefficients (**Supplementary Table S3**) or pathological lesions in livers, kidneys, and spleens (data not shown).

## Discussion

Lead is a toxic heavy metal that can induce a range of adverse health effects in humans. The body burden of Pb in the general population has been estimated to be 1,000 times higher than that of prehistoric humans (Patterson et al., 1991). In the last 10 years, the world consumption of Pb has continued to increase, and developing countries are especially facing serious Pb pollution problems (Zhai et al., 2015). Once accumulated in the human body, Pb is hard to excrete, and its biological half-time can be several decades (Sugita, 1978). This is confirmed by our results, as the Pb levels in mice of the Pb-only group did not decrease even after exposure had been stopped for 9 weeks (**Table 3**). Also noted was the lack of effectiveness of excipients including microcrystalline cellulose, low-substituted hydroxypropyl cellulose, and silicon dioxide against Pb toxicity, indicating the necessity to develop potential dietary strategies.

In the present study, nutrients including essential elements, vitamins, plant extracts, and probiotics were selected and incorporated into the design of two different dietary supplements. Both DSA and DSB could significantly reduce Pb levels in the tissues and blood of mice (**Table 3**). The protection offered by the former supplement may be due to the Pb-chelating capacities of catechins and *L. plantarum* CCFM8661 (Chen et al., 2002; Yin et al., 2016). Grape seed extracts, tea polyphenols, and probiotics can also protect the intestinal barrier, which may in turn inhibit Pb absorption in the gut (Watson et al., 2004; Goodrich et al., 2012; Zhai et al., 2016). Based on previous studies, essential metals, and vitamin C supplementation could prevent heavy metal absorption and decrease Pb levels in humans and animals (Goyer and Cherian, 1979; Zhai et al., 2015). This may explain the protective effects of DSB supplementation against the increase of Pb in the tissues and blood of mice.

Lead accumulation can trigger abnormal oxidative stress and in turn induces histopathological alterations in the tissues, which is a main mechanism of Pb toxicity (Stohs and Bagchi, 1995). Downstream protective effects of Pb removal from the body by the two dietary supplements included recovery of the alterations in oxidative stress-related parameters in the livers, kidneys, and brains of mice (**Tables 4**–**6**). Moreover, nutrients including vitamin C, grape seed extract, tea polyphenols, and probiotics have been reported to be natural anti-oxidants (Noroozi et al., 1998; Bagchi et al., 2000; Zhai et al., 2014), which may offer direct protection against Pb-induced oxidative stress. Zn functions as a cofactor of Cu/Zn SOD, and the supplementation of this essential metal may also play a role in the alleviation of oxidative stress (Prasanthi et al., 2010).

Hematological and nervous systems are key targets in the progression of Pb toxicity. ALAD is a vital enzyme in the hematological system, which is sensitive to Pb exposure (Wang and Fowler, 2008). The Pb-induced inhibition of ALAD damages heme synthesis and increases the level of ZPP, which further affects the levels of RBCs and hemoglobin in the blood (Alexander et al., 1998). In the present study, oral administration of DSA and DSB significantly recovered both of these hematological indicators (**Figures 1**, **2** and **Table 7**), indicating protection of both dietary supplements against Pb-induced dysfunctions in the hematological system. Ca and Zn have been reported to alleviate Pb-induced neurotoxicity by competing for the binding sites of enzymatic proteins in the nervous system (Prasanthi et al., 2006). Vitamin C, procyanidins and tea polyphenols can inhibit the production of reactive oxygen species induced in the nervous system by Pb, which further reverses the effects of hippocampal injury and reduces neuronal cell apoptosis in animals (Liu et al., 2014; Ebrahimzadehbideskan et al., 2016). Moreover, recent clinical studies confirmed that probiotics may play a role in alleviating nerve dysfunctions via the “brain-gut axis” (Pinto-Sanchez et al., 2017). These analyses can explain the marked improvement of the learning and memory abilities of the Pb-exposed mice after the DSA and DSB treatments.

We noticed that both DSA and DSB offered superior protection against Pb toxicity over that of any single component of the dietary supplements. Compared with a single *L. plantarum* CCFM8661 treatment (Tian et al., 2012), DSA and DSB were more efficient in recovering antioxidant enzyme activities and inhibiting lipid peroxidation in the tissues of mice. This may be due to the antioxidative stress role of vitamin C and plant extracts in the dietary supplements. Compared with the supplementation of micronutrients, catechins, or procyanidins alone (Patra et al., 2001; Zhang et al., 2004; Liao et al., 2008; Yin et al., 2008), the dietary supplements in the present study provided more significant protective effects to reduce the Pb burden in the mice. This may be explained by a synergistic effect of multiple nutrients from DSA and DSB to increase Pb excretion and prohibit Pb absorption. Also of importance is that besides the above-mentioned protection against Pb toxicity, these dietary supplements may provide other beneficial effects for humans such as the supplementation of essential metals, scavenging of free radicals, and modulation of the gut microbiota.

The intervention and therapy groups in the present study were designed to understand the optimal therapeutic strategy for the dietary supplements. Taking all effects into consideration, DSA and DSB treatment in the intervention groups offered superior protective effects on decreasing Pb levels in the tissues (livers and brains) and recovering the learning and memory abilities of mice than those in the therapy groups (**Tables 3**, **8**). As the dietary supplements in the intervention groups were administered during the exposure to Pb, they may inhibit the initial absorption of Pb in the gut, thus preventing the accumulation of this metal in the tissues and alleviating downstream adverse effects including pathological injuries and cognitive disorders. If administered after exposure (therapy groups), the dietary supplements seemed to be less effective as Pb had already accumulated in the body and triggered a range of physiological dysfunctions.

Dimercaptosuccinic acid is a chelator commonly used for the treatment of Pb toxicity (Chisolm, 2000). Compared with the DMSA treatment, the dietary supplements did not exhibit the same protection in reducing the Pb burden (**Table 3**) and recovery of the ZPP level (**Figure 2**), but they did show more obvious alleviation of oxidative stress (**Tables 4**–**6**) and cognitive dysfunctions (**Table 8**). This may indicate that the dietary supplements designed in the present study may offer a route of protection against Pb toxicity besides metal chelation. The anti-oxidant and neuroregulation properties of the nutritional components may be involved in this mode of protection. Safety evaluations are essential for the development of novel dietary supplements (Kruger and Mann, 2003). The nutrients selected in the present study have been widely used in food and dietary supplements. The acute toxicity test and 30-day feeding trial also confirmed that the consumption of DSA and DSB at different doses did not induce physiological disorders in mice, indicating that these dietary supplements appeared to be relatively safe. Further clinical safety assessments are needed to evaluate their application in humans.

## Conclusion

This study showed that the probiotics, micronutrients, and plant extracts contained in the tested dietary supplements appear to have protective effects against Pb toxicity in mice. The supplements could decrease tissue Pb accumulation, alleviate tissue oxidative stress and recover dysfunctions in the hematological and nervous systems of mice. The animal safety assessment confirmed that these dietary supplements barely induced any side effects in the studied mice.

## Author Contributions

QZ and LY carried out the experiments and drafted the manuscript. JZ and HZ participated in the analysis of the data. FT conceived of the study and managed the project design. WC helped to revise the manuscript. All authors read and approved the final manuscript.

## Conflict of Interest Statement

The authors declare that the research was conducted in the absence of any commercial or financial relationships that could be construed as a potential conflict of interest.
