# Supplementary material for: Protective Effects of Dietary Supplements Containing Probiotics, Micronutrients, and Plant Extracts Against Lead Toxicity in Mice
Source: Front Microbiol. 2018 Sep 11;9:2134. doi: 10.3389/fmicb.2018.02134 (PMC6141689; doi:10.3389/fmicb.2018.02134)
Supplement: Supplementary file 1 [file Table_1.DOCX]

Table S1 Effects of dietary supplements on the levels of routine hematological indicators in the serum of male mice (30-day feeding trial for safety evaluation)

| Groups | Red blood cells  (10^12^/L) | Hemoglobin  (g/L) | White blood cells  (10^9^/L) |
| --- | --- | --- | --- |
| Control | 10.13±0.54 | 154.00±7.00 | 4.11±0.08 |
| Low-dose DSA | 10.04±0.38 | 155.00±4.36 | 4.09±0.23 |
| Mid-dose DSA | 10.17±0.21 | 153.33±3.21 | 4.12±0.15 |
| High-dose DSA | 10.14±0.14 | 154.00±7.00 | 4.20±0.13 |
| Low-dose DSB | 10.05±0.58 | 153.67±4.62 | 4.14±0.16 |
| Mid-dose DSB | 10.17±0.39 | 152.67±0.58 | 4.19±0.21 |
| High-dose DSB | 10.29±0.41 | 155.33±3.06 | 4.12±0.12 |

Values are for 10 mice per group. No significant differences were observed within each row comparison.
