# Supplementary material for: Protective Effects of Dietary Supplements Containing Probiotics, Micronutrients, and Plant Extracts Against Lead Toxicity in Mice
Source: Front Microbiol. 2018 Sep 11;9:2134. doi: 10.3389/fmicb.2018.02134 (PMC6141689; doi:10.3389/fmicb.2018.02134)
Supplement: Supplementary file 2 [file Table_2.DOCX]

Table S2 Effects of dietary supplements on the levels of biochemical parameters in the blood of female mice (A thirty-days feeding trial for safety evaluation)

| Groups | ALB  (g/L) | ALT  (U/L) | AST  (U/L) | BUN (mmol/L) | CRE (μmol/L) | TCHO (mmol/L) | TG (mmol/L) | TP  (g/L) |
| --- | --- | --- | --- | --- | --- | --- | --- | --- |
| Control | 21.63±1.25 | 30.07±4.63 | 116.56±8.48 | 12.59±1.59 | 35.73±4.28 | 1.77±0.17 | 0.46±0.05 | 63.95±3.93 |
| Low-dose DSA | 21.41±1.99 | 30.14±3.65 | 112.42±11.03 | 13.33±1.19 | 34.60±3.11 | 1.76±0.16 | 0.47±0.07 | 64.67±3.98 |
| Mid-dose DSA | 20.81±1.52 | 29.58±4.35 | 113.03±14.67 | 13.71±2.08 | 35.43±2.70 | 1.81±0.17 | 0.46±0.07 | 64.68±3.07 |
| High-dose DSA | 20.69±2.04 | 31.46±5.10 | 111.10±14.11 | 12.95±1.49 | 36.02±1.76 | 1.68±0.10 | 0.45±0.05 | 64.41±4.01 |
| Low-dose DSB | 21.38±1.64 | 31.57±4.51 | 113.08±13.31 | 12.15±1.49 | 34.29±3.28 | 1.81±0.11 | 0.45±0.06 | 66.36±3.52 |
| Mid-dose DSB | 22.53±1.60 | 29.75±4.44 | 116.98±9.17 | 13.32±1.85 | 33.42±2.74 | 1.83±0.18 | 0.46±0.05 | 64.67±3.40 |
| High-dose DSB | 22.36±1.13 | 31.14±3.71 | 114.25±9.89 | 12.44±2.28 | 35.23±2.31 | 1.79±0.17 | 0.48±0.04 | 64.35±3.62 |

Values are for 10 mice per group. No significant differences could be observed within each row comparison. ALB, albumin. ALT, glutamic pyruvic transaminase. AST, glutamic oxalacetic transaminase. BUN, blood urea nitrogen. CRE, creatinine. TCHO, total cholesterol. TG, triglyceride. TP, total protein.
