# Supplementary material for: Protective Effects of Dietary Supplements Containing Probiotics, Micronutrients, and Plant Extracts Against Lead Toxicity in Mice
Source: Front Microbiol. 2018 Sep 11;9:2134. doi: 10.3389/fmicb.2018.02134 (PMC6141689; doi:10.3389/fmicb.2018.02134)
Supplement: Supplementary file 3 [file Table_3.DOCX]

Table S3 Effects of dietary supplements on organ coefficients in male mice (30-day feeding trial for safety evaluation)

| Groups | Liver weight/Body weight (%) | Kidney weight/Body weight (%) | Spleen weight/Body weight (%) |
| --- | --- | --- | --- |
| Control | 3.17±0.12 | 0.49±0.04 | 0.29±0.01 |
| Low-dose DSA | 3.25±0.17 | 0.49±0.02 | 0.28±0.03 |
| Mid-dose DSA | 3.27±0.19 | 0.49±0.03 | 0.29±0.02 |
| High-dose DSA | 3.28±0.17 | 0.50±0.04 | 0.28±0.02 |
| Low-dose DSB | 3.25±0.10 | 0.48±0.03 | 0.27±0.02 |
| Mid-dose DSB | 3.21±0.12 | 0.49±0.04 | 0.28±0.03 |
| High-dose DSB | 3.21±0.15 | 0.48±0.03 | 0.28±0.03 |

Values are for 10 mice per group. No significant differences were observed within each row comparison.
